# Two state model for a constant disease hazard in *paratuberculosis* (and other bovine diseases)

**DOI:** 10.1186/s13567-015-0189-9

**Published:** 2015-06-19

**Authors:** Yoram Louzoun, Rebecca Mitchell, Hilla Behar, Ynte Schukken

**Affiliations:** Gonda Brain Research Center and Department of Mathematics, Bar-Ilan University, Ramat Gan, Israel; ASM Post Doctoral Fellow at Centers for Disease Control and Prevention, Atlanta, Georgia; GD Animal Health, Deventer, the Netherlands; Department of Population Medicine and Diagnostic Sciences, Cornell University, Ithaca, NY USA

## Abstract

Many diseases are characterized by a long and varying sub-clinical period. Two main mechanisms can explain such periods: a slow progress toward disease or a sudden transition from a healthy state to a disease state induced by internal or external events. We here survey epidemiological features of the amount of bacteria shed during *Mycobacterium Avium Paratuberculosis* (MAP) infection to test which of these two models, slow progression or sudden transition (or a combination of the two), better explains the transition from intermittent and low shedding to high shedding. Often, but not always, high shedding is associated with the occurrence of clinical signs. In the case of MAP, the clinical signs include diarrhea, low milk production, poor fertility and eventually emaciation and death. We propose a generic model containing bacterial growth, immune control and fluctuations. This proposed generic model can represent the two hypothesized types of transitions in different parameter regimes. The results show that the sudden transition model provides a simpler explanation of the data, but also suffers from some limitations. We discuss the different immunological mechanism that can explain and support the sudden transition model and the interpretation of each term in the studied model. These conclusions are applicable to a wide variety of diseases, and MAP serves as a good test case based on the large scale measurements of single cow longitudinal profiles in this disease.

## Introduction

Mycobacterium Avium subspecies Paratuberculosis (MAP) is characterized by a long sub-clinical period. Cows are typically infected early in life, but show clinical signs of disease only a number of years later. Such a sub-clinical to clinical transition is observed in many human and animal diseases, including, among many others, Human Immuno-deficiency Virus (HIV) infections [1], Herpes virus infections [2-4], prion induced diseases [5], Mycobacterium bovis [6], Mycobacterium tuberculosis infections [7] and Bovine Leukemia Virus (BLV) infections [8]. The observed transition from sub-clinical to clinical disease can be interpreted in two main ways: either the disease is slowly aggravating over time, and the observed clinical symptoms are simply the end-point of a slow deterioration process, or the sub-clinical period is indeed latent, and some event led to instability and the eruption of clinical disease.

Different infectious diseases have been characterized to show in clinical progression one of the two scenarios defined above. Most chronic diseases, such as Hepatitis C Virus (HCV) and HIV show slow progression to a clinical stage (Acquired Immune Deficiency Syndrome (AIDS) in HIV and cirrhosis in HCV) [9]. However, some such as Herpes infections show no progression until either an external or internal event (other diseases, fatigue, pregnancy, stress…) leads to a clinical stage [10]. Note that Herpes Simplex Virus (HSV) has a relatively short clinical period and is controlled within a relatively short period of time.

The classification of infectious diseases into these two general categories has important therapeutic implications. If a disease belongs to the slowly progressing group, then treatment before the clinical stage can prevent or delay further deterioration. If on the contrary the transition to the clinical stage is sudden, the optimal scheme to prevent clinical disease would be prevention of events that can induce the transition to the clinical stage.

This distinction also has implications for the predictability of future stages of clinical disease. In a slowly progressing disease, the probability of future clinical signs can be estimated from the position in the path to a full blown disease; while in a sudden transition, the probability for disease should be estimated by the frequency of events that can induce such a transition.

Infections of dairy cows with MAP are generally assumed to occur at a very young age [11]. Young calves show the presence of so-called peyers’ patches that allow the early uptake of MAP bacteria. The MAP infected calves remain sub-clinical for years, or even lifelong. Clinical signs of Johne’s disease, the clinical stage of MAP infections, usually occur in adult cattle [12]. The onset of clinical signs often, but not only, occurs after giving birth.

In order to evaluate which of these two disease progression phenomena is most likely to occur with MAP infections, we propose a relatively simple mathematical model, the transition model, and compare it to a set of observations regarding infection and disease dynamics. We suggest here that a similar approach, assuming the presence of similar longitudinal data, may be applied to other infectious diseases.

In the following section we discuss a set of observations, and then compare multiple models to explain these observations:

A) A Markov model with three different possible states.
B) A deterministic model for the growth of the bacterial population.
C) Different versions of a stochastic dynamics as described by Stochastic Differential Equations (SDE).

While all models presented here are simplistic models, they can serve to differentiate between general scenarios. Given the complexity of any disease and the number of free parameters that can be introduced in mathematical models, we can almost always find a complex enough model that could explain a set of observations. We intend to show here that a very simple model can explain multiple observed phenomena depending on the choice of parameters and the value of the chosen parameters. Therefore the model proposed here aims to be a flexible yet realistic model describing real life phenomena.

## Materials and methods

### Ordinary Differential Equation (ODE) solution

The ODE were solved numerically using Matlab fourth/five order Runge Kutta, as applied in the MATLAB, ode45 function assuming non-stiff equations.

### Stochastic Differential Equation (SDE) solution

The SDE is modeled as an ODE with Ito noise unless stated otherwise. It was solved using Matlab when after each step an Ito noise is calculated. Specifically, a normal random variable with a zero mean and a variance of *σ*^2^*dt* was added in each step of the ODE solution to simulate a Wiener process [13], where *dt* is the time step size. The ODE was first solved using a fourth order Runge Kutta methods [14]. Then, noise was added.

### Markov model

The Markov models were solved numerically using Matlab, where the probability for each cow to die is taken from a binomial distribution. The initial number of the cows was 1000, and the probability to die was set to 0.001.

### Stylized observations in MAP epidemiology

In order to study the transition to the disease state, we analyzed three farms with natural infections, and examined the time course of bacterial shedding for over 1000 cows. Note that experimental and natural infections vary in many aspects [15]. The current analysis is only focused on the dynamics of natural infections. A detailed description of the datasets used is found in the accompanying manuscript [15]. We here provide a short description of the observation.

Data for this study were gathered from three longitudinal field studies, one longitudinal follow up in an experimentally aggregated population and multiple experimental infection trials.

- Field study 1 comprised three dairy farms (100, 150, 300 lactating animals per farm) in the Northeastern US [16]. Animals in study 1 were sampled twice per year by fecal culture and four times per year by ELISA for seven years after initial farm enrollment. For details of study design, sample collection and preliminary data processing, see previously published work [16-18].
- Field study 2 followed animals on a single dairy farm with approximately 100 lactating Guernsey breed cattle in Pennsylvania (US) for a period of 20 years during an intervention program. Details of farm size, MAP prevalence and study design are available in previously published work [19]. Animals in this population were tested semi-annually by fecal culture.
- Field study 3 followed animals on 17 Dutch dairy farms (32 to 104 animals per farm with a total of 1072 cows) during a national monitoring program over the course of 3.5 years. Animals were tested by ELISA and fecal culture at 6 month intervals [20].

In order to simplify the analysis, we defined for each cow three possible states: A) Non Shedding, B) Mild shedding, and C) High shedding. We defined the last stage to be any value above or equal to 50 Colony-Forming Units (CFU) per gram of feces, and the mild stage to be between 1 and 50 CFU per gram of feces. Generally, cows that are shedding high numbers of bacteria show or will show clinical signs of Johne’s disease.

In the current analysis, shedding time-series had typical intervals of 90–180 days, and the vast majority of cows (94.5%) never reached high shedding. In the cows that never reached high shedding (189/3397 ~ 5.5%), the vast majority of cows (>90%) never went back to mild or low shedding and had high shedding values until they were removed from the herd due to culling or death.

A large fraction of the cows never presenting high shedding levels may actually have been infected at least for some time. Among the cows never producing high shedding levels, 10% had some evidence of infection (Blood/Milk Enzyme-Linked ImmunoSorbent Assay (ELISA), tissue samples, or intermittent low or fluctuating intermediate levels of shedding).

**Figure 1:**
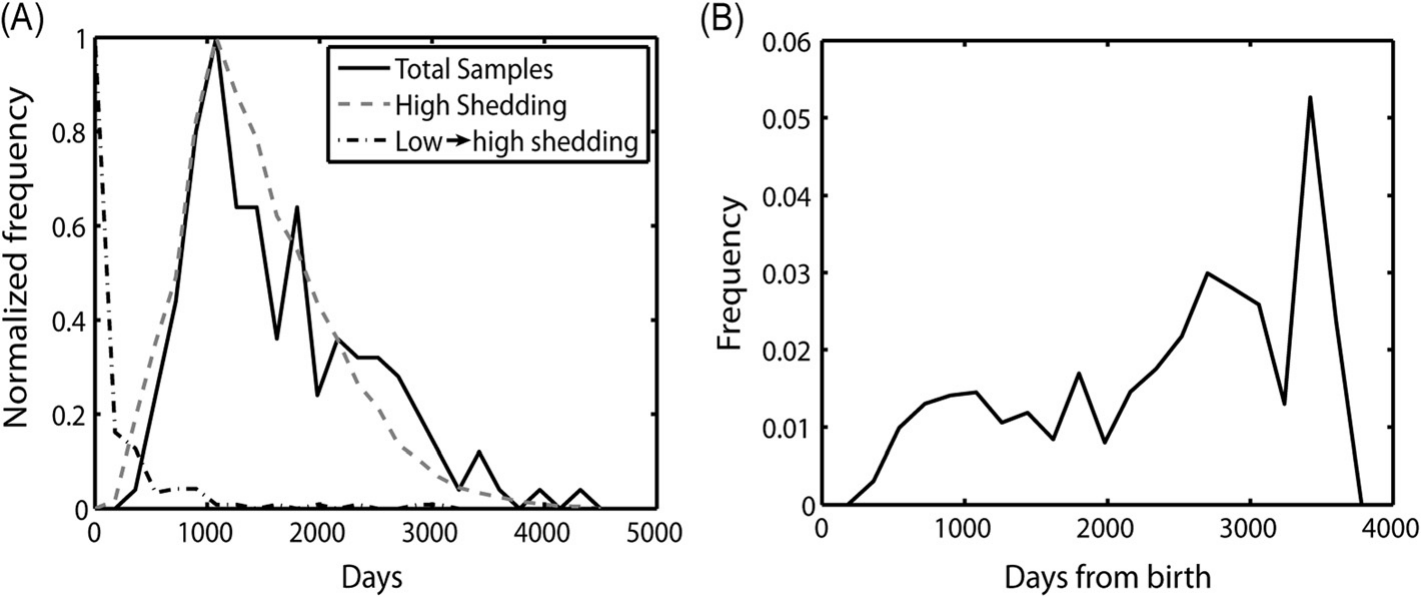
**Experimental results.** **(A)** Total fraction of observed cows in all farms studied (full line) as a function of the cow age, and the fraction of cows showing first clinical signs as a function of cow age (gray dashed line). The black dashed dotted line is the fraction of cows showing clinical signs as a function of time since first shedding (early shedding is not included in this analysis). **(B)** The fraction of cows getting infected from the cows that are still in the herd as a function of the cows age.

Some of the cows show an initial low shedding stage before moving on to high shedding values. However, the average time from the first non-zero level to high shedding is one sample (less than 180 days) with a narrow distribution (Figure 1A, dashed dotted black line). This distribution was probably an upper limit, since given the long time difference between sampling points, the transition may actually have been much faster than the time between two measurement points.

Before high shedding started, the fraction of cows expressing a first high shedding event from the cows that were still in the herd at a given age was computed, by dividing the fraction of cows expressing first high shedding at a given time point (Figure 1A, dashed gray line) with the number of samples taken at the same time (Figure 1A, full black line). This ratio increased until it was stabilized at day 1000 (approximately 3 years of age). From there on, it remained approximately constant for several thousand days (Figure 1B). Beyond 3000 days, observations become scarce and the ratio was noisy.

One can thus summarize the epidemiology of MAP by the following stylized facts:

I. Most MAP infected cows never reach high shedding.
II. Within the MAP infected cows reaching high shedding, the vast majority of cows never go back to low/no shedding.
III. In the group of cows that are high shedders, these animals reached the stage of high shedding fast after initial shedding, compared to the length of the sub-clinical period.
IV. The ratio to reach high shedding is constant after approximately an age of 1000 days.
V. Most cows are infected and some occasionally shed low levels of bacteria.

We have here equated clinical signs to high shedding levels of MAP. While such shedding levels are often seen in cows with clinical signs defining Johne’s disease, the presence of high shedding is not completely equivalent to a transition to a clinical stage. Still, a clear relation between high-shedding and clinical signs has been reported. A much more detailed description of the epidemiology and clinical signs can be found in the accompanying paper by Mitchell et al. [15]. More details on the sampling scheme in the herds can be found in Schukken et al. [18].

### Transition model

**Figure 2:**
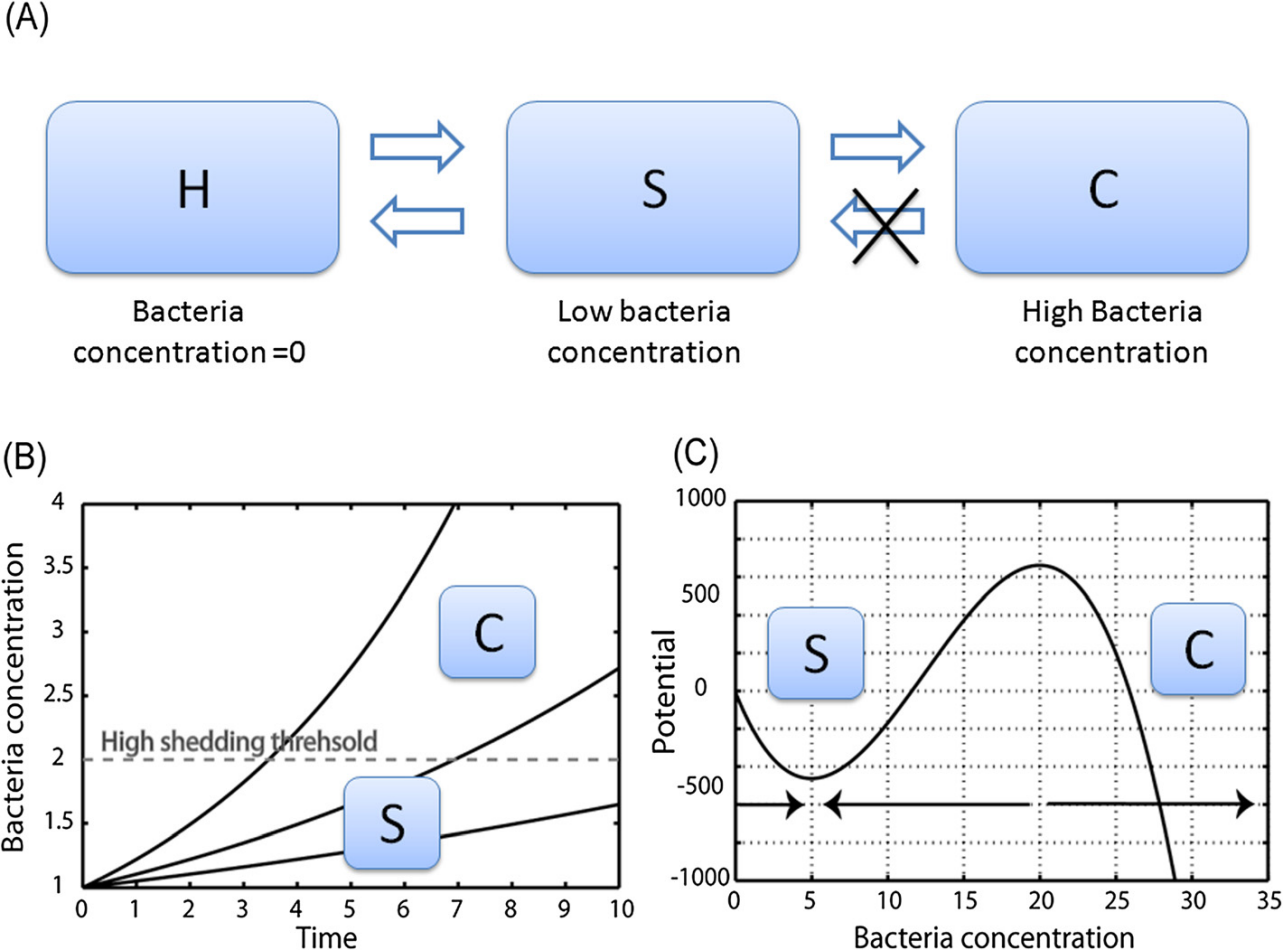
**Descriptions of the different models.** **(A)** Markov model of disease dynamics with three states: uninfected (H), Sub-clinical (S) and cows showing clinical signs (C). The observations seems to show a unidirectional dynamics, where the empty arrows do not exist in reality or have a very low probability. **(B)** Deterministic model of bacteria concentration growth (full lines) eventually leading to the transition of a threshold (dashed gray line) and to clinical signs. **(C)** Dynamic model producing two states with a potential (full line) that has two attractors. The left attractor is the sub-critical stage and the right attractor is the clinical stage (i.e. the stage where clinical signs are exposed). In this case the transition between the two states is through random fluctuations.

The epidemiology of MAP as described above can be represented as a three state model: The first state is healthy, uninfected (H). The second state is sub-clinical with potential low or intermediate shedding (S) and the third state is high shedding with potentially signs of clinical disease (C). The transitions in this model would be from H to S and possibly back to H and from S to C, with no possible transition back from C to S (Figure 2A). Within such a model two scenarios are possible: Either the transition is stochastic; leading to a variance between the times it takes different cows to move to the C state, or transition is deterministic, with a slowly deteriorating state ending with the transfer to the clinical state (Figure 2B). In the latter model, the difference between the times that cows reach state C is either in the initial condition or in the parameters of the disease.

In order to compare the two models, we propose a generic ODE and SDE framework to study the parameters required by each type of model and to determine which model is more plausible.

### Markov model

**Figure 3:**
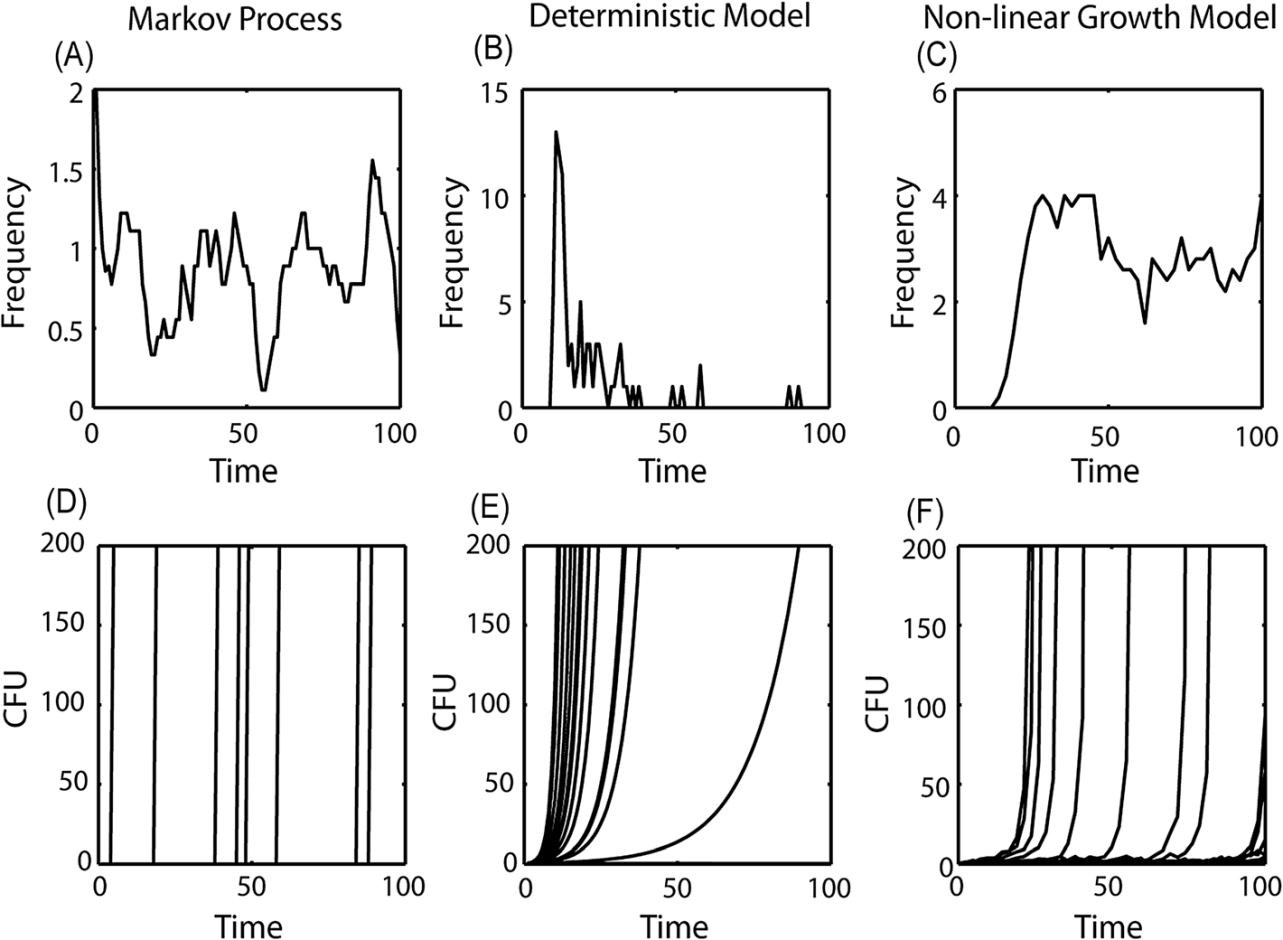
**The behavior of the different models as a function of time.** The first line represents the frequency of cows becoming sick at a given time point (x axis) **(A)** for the first model- Markov process, **(B)** for the second model- the deterministic model, and **(C)** for the non- linear growth model. The second line represents the x values (the bacteria level within a given cow) as a function of time for some cows, **(D)** for the Markov process, **(E)** for the deterministic model and **(F)** for the non- linear growth model.

A Markov model can reproduce many of the observed features. The fraction of cows that reach high shedding is determined by *p*(*S* → *C*), which can be pre-defined to be very low. The absence of cows that heal simply represent the fact that *p*(*S* → *C*) is practically 0. The constant ratio is explicitly built into this model, and the low level of shedding of most cows can be obtained by setting *p*(*S* → *H*) to be very low (Figure 2A). However, it fails to reproduce the sensitivity to the dose used to infect cows. In the simulations of this model each cow that is in state S goes to C with a probability of *p*(*S* → *C*). Cows in state C cannot go back to state S. While in natural infection circumstances, the total fraction of infected cows is usually below 30%. In high-dose infection experiments, the fraction of cows showing high shedding and clinical signs in high-dose experimentally infected animals reaches almost 100% (accompanying paper by Koets et al. [21]). Another weakness of the Markov model is its failure to explain the rarity of clinical diseases in the first two years of MAP infection, although the vast majority of MAP infected cows are infected in the first 360 days of their life (Figures 3A and 3D).

One could amend these two weaknesses by explicitly incorporating the difference between experimental and natural infections into the model, and assuming that the transition probabilities are determined by environmental and internal elements. In such a model, the transition probabilities would be much higher for experimental than for natural infections. The lack of an initial refractory period can be amended by introducing a larger number of intermediate stages between the S and C stages.

Given enough such intermediate stages, the Markov chain behavior approaches a random variable. Thus, while technically correct, we will show that a random variable description provides a simpler description of such a stochastic process.

### Deterministic immune control model

The second model can be studied using a standard ODE approach, since it does not contain stochastic elements. The simplest model would be constant reproduction and destruction rates for the bacteria in a single cow. For the sake of simplicity, let us model the bacteria level within a given cow, and denote it by x. Let us assume that the bacteria are destroyed by the immune system or cleared by any other mechanism at a rate of *δ*, and grows at a rate of *v*, with a net difference of *β* = *v* − *δ*. If this is the only interaction, the dynamics are determined by the linear equation:

1 $$ x\hbox{'}=\beta x $$

with the exponential solution of:

2 $$ x(t)=x(0){e}^{\beta t} $$

In this model, only two solutions would be possible: either the bacteria are cleared from the host, or the bacteria are growing exponentially and the high shedding occurs probably with the onset of the clinical signs. We do not explicitly state the properties of the bacterial dynamics, once high shedding is reached, but the dynamics at this stage have no significant effect on the conclusions, since we assume that once this high shedding is reached, the cow cannot go back to the transient or healthy state. A simple description of the dynamics beyond this stage can be through logistic growth:

3 $$ x\hbox{'}=\beta x-\sigma {x}^2, $$

where *β* = *v* − *δ*, as in Equation (1), and *σ* is the competition rate of the bacteria. The values of *σ* are low enough (Figure 2B).

For negative values of *β*, the cow will stay healthy all its life. For positive values of *β*, the time to reach the onset of clinical signs would be proportional to 1/*β*. In such a model, we would have to assume that in the majority of the population the value of *β* is negative and in a small part of the population the value of *β* is positive. Such a simple model would represent a model where either the bacteria or the host are predisposed to induce clinical signs, or no disease can occur.

Such a model is inconsistent with multiple observations:

A) In this model we do not expect cows not eventually becoming ill to have bacteria in them after some stage, since the bacteria frequency is expected to decrease over time in these cows.
B) The ratio is not expected to be approximately uniform in time, since there is no *apriori* reason to assume that 1/*β* would be distributed uniformly for all positive *β* values (see for example the result for a uniform distribution of *β* in Figures 3B and 3E).
C) In this model, the disease probability is not affected by the initial bacterial dose during infection, in contrast with the clear difference in the frequency of sick cows in experimental and natural infection studies, as mentioned above.

### Non-linear model

The two approaches can be combined through a slightly more complex model that includes two realistic features. The first feature to include is an explicit non linear growth rate in addition to the elements above. The power of the non-linear growth rate can be any power above one. We here use a power of two for the sake of simplicity. This would represent a positive feedback of the bacteria on itself. Such a feedback can occur if for example, bacteria survive better within granuloma, which in turn are produced by the bacteria. The model would then become:

4 $$ x\hbox{'}=-\beta x+{x}^{\gamma };\gamma =2 $$

Note that many different positive feedback loops can produce a similar behavior, beyond the possible effect of granuloma.

In contrast with the model of Equation (1), this model can exhibit a transition to a disease even if *β* is positive, if the initial value of *x* is higher than − *β*. This model is basically equivalent to the previous model with the twist that a cow that would not have become ill in the model of Equation (1) will become clinically ill, if it is infected with a high enough dose of bacteria. This seems to be in agreement with reality, where experimentally high-dose challenged cows have a much higher probability of presenting high shedding and clinical signs than naturally infected ones.

However, this model still suffers from two problems discussed for the model in Equation (1), namely:

A) In this model we do not expect cows not eventually becoming ill to have bacteria in them, since the population that will never be sick has low *x* values, and in this domain, Equations (1) and (4) are similar.
B) The ratio is not expected to be uniform, since there is no *apriori* reason to assume that 1 / *β* would be distributed uniformly. The non-linear term leads to the divergence of the model in a finite time, and as such would have a very limited effect on the spread of times to high shedding.

### Stochastic transition model

These two limitations can be solved using two slight modifications to the model: the introduction of a constant source of bacteria (*A*) and the introduction of fluctuations in the bacteria levels through a random noise term in the bacterial dynamics, leading to the following Stochastic Differential Equation (SDE):

5 $$ x\hbox{'}=A-\beta x+{x}^2+\sigma \varepsilon (t)x, $$

where *ε*(*t*) is a normal random variable with a noise level *σ*. The constant source of bacteria can represent a reservoir of bacteria produced immediately following infection that releases bacteria to the blood or the gut [22]. The noise term represents random fluctuation representing the effect of an internal or external event (weather, diseases, pregnancies, diet etc.) on the bacteria.

**Figure 4:**
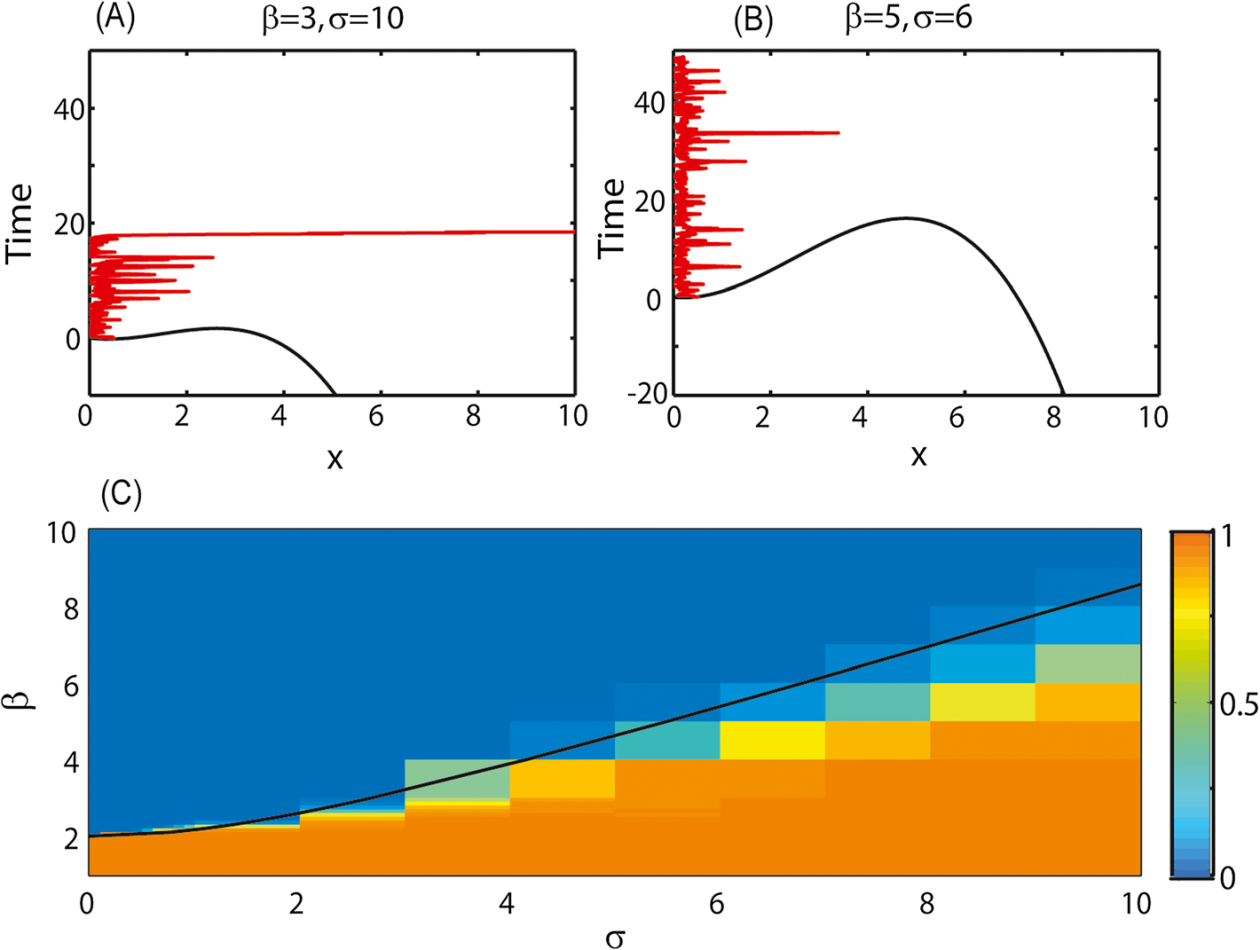
**The behavior of the stochastic transition model for different parameter values.** **(A)** and **(B)** potential barrier for different parameter values (black lines) and the resulting dynamics (red lines). Time is on the y axis and x values are on the x axis. For low *β* and high *σ* transition to high shedding will be very rapid (A), while for high *β* and low *σ* it may never happen (or may take a very long time) (B). **(C)** Fraction of cows reaching high shedding at t = 1000. For high *σ* and low *β* the fraction is close to 1 (orange), while for low *σ* and high *β* the fraction is close to zero (blue). There is an intermediate region, where a limited fraction of cows becomes high shedders. The black line represents the parameter values that equal to the distance between the low attractor and the unstable fixed point.
- Within the cows reaching high shedding, the vast majority of cows never go back to low/no shedding. This asymmetry in the transition is a result of the different properties of the two attractors. When the system resides in the low bacterial level attractor, it has a constant probability of moving to the high attractor, and a large enough fluctuation is enough to move it to the high attractor. The opposite is not true. When the system resides at the high attractor, it will diverge in a finite short time, and will thus never be able to return to its initial state.
- The third observed feature is the fast emergence of clinical signs after initial shedding. Such a fast growth is indeed expected from the non-linear growth term, which as mentioned above will lead a finite time divergence of *x*.
- The non-zero value of *A* prevents the system from falling to the *x* = 0. Thus, in this model, every cow that was infected will only become uninfected again if *A* = 0.
- The ratio to reach high shedding is constant after a period and then slightly deteriorates (Figures 3C and 3F).

For the appropriate parameter values (as will be further discussed), this model has two attractors: a low shedding attractor determined by the value of *A*, and a high shedding level, attractor at infinity (Figure 2C). The noise level *σ* determines the probability to move from the low attractor to the high one. Within this parameter range, this model does indeed produce all the stylized facts mentioned above:

- If *β* is high enough and the noise level *σ* is low enough, most cows will never reach high shedding, unless a very high dose is introduced as might happen in the case of high-dose experimental challenge infections (Figures 4B and 4C).

While this model explains most observed features, it has one weakness, which is sensitivity to the value of the *β* parameter. To test the validity range of this model, we performed a sensitivity analysis of the model.

### Parameter sensitivity

The following two sections are quite mathematical and the biological conclusions of the paper can be understood without them. We here performed a sensitivity analysis to the results of Equation (5) and explained the results. The dynamics of Equation (5) are determined by the values of *A*, *β* and *σ*. For any non-zero value of *A*, the bacterial level will always remain positive. However, beyond this direct effect, the contribution of *A* can be scaled into the other parameters, by changing, $x\to x/\sqrt{A},t\to \sqrt{A}t$ to obtain:

6 $$ x\hbox{'}=1+\left[\sigma \varepsilon (t)-\beta \right]x+{x}^2, $$

where *β*, *σ* have been rescaled. There are, up to a scaling factor, only two real free parameters in this system. In the absence of the noise (*σ* = 0), Equation (6) can have either a single attractor at infinity or two attractors, one at infinity and one at $\frac{\beta }{2}\left[1-\sqrt{1-4/{\beta}^2}\right]$. The two attractors solution can only occur if *β* > 2. Thus, with a weak immune response (low value of *β*), all cows will become rapidly high shedders regardless of the parameter *σ*. For a strong immune response (high value of *β*), there is a range of *σ* where only a few cows become high shedders within a reasonable amount of time since the moment the animals became infected with MAP.

In order to understand the relation between the probability to get infected and the parameters *β* and *σ*, the dynamics of *x* can be rewritten as:

7 $$ x\hbox{'}=-\frac{dV(x)}{dx}+\sigma \varepsilon (t), $$

where *V*(*x*) is the potential limiting *x* to be in the low attractor. Assuming *x* is close to the minimum potential, the size of *σ* must be similar to the distance between the low attractor and the unstable fixed point. The *σ* value equal to this distance is denoted by a black line in Figure 4C. If *σ* is much smaller than this distance, we expect the average time to express high shedding and clinical sign to be high, while if it is larger than this distance, this time to high shedding will be low.

In order to check that this is the case, we simulated the dynamics in Equation (6) for different *σ* values, and computed the average time to high shedding and clinical signs (Figure 4C). As expected, a sharp transition occurs near the black line, where the *σ* value is equal to the distance between the low attractor and the unstable fixed point. The dynamics at the two sides of this line are exemplified in Figures 4A and 4B respectively. One can clearly see from Figure 4C that for any *σ* value, the range of *β* value where the transition probability is neither too low nor too high is limited. This is the main caveat of this proposed model.

### Non uniform beta distribution

As mentioned above, in the rescaled units *β* and *σ* should be of the same order for a finite yet not too large transition to clinical signs probability to emerge. This can obviously be tuned into the system. However, since *β* represents the immune response, which is affected by a large number of factors, there is no *a priori* biological reason that these parameters should have a similar range.

**Figure 5:**
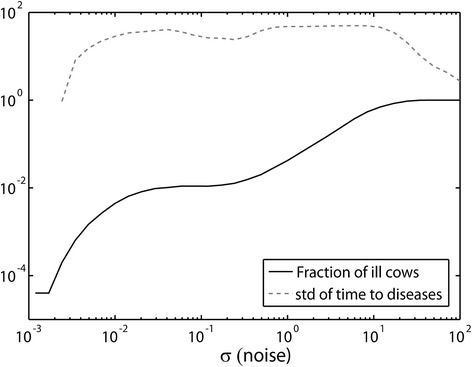
**Fraction of cows and std of time to disease as a function of noise level.** Fraction of cows reaching high shedding as a function of the noise level *σ* in the model with a wide *β* distribution (black line), and the standard deviation of the time it takes to reach an ill state (gray dashed line). The simulations were run on a scale of 100 time units in arbitrary units. One can see that for a wide range of *σ* values (two orders), the fraction of cows getting ill is constant and low and the standard deviation of the time to reach sickness is high. Thus the model is not limited to a precise value of *σ* or *β* to reproduce the observed dynamics.

However, one can assume that *β* has a distribution in the population and that *β* varies among cows. Assume for example that *β* has a uniform distribution between 2 and 10. As was mentioned earlier, for values of *β* below 2, the cows become sick with a probability of 1. Moreover, the transition will be very rapid. The cows with high *β* values will never be sick, even for high noise levels and thus will not be observed as sick cows, only the cows with *β* values close to 2 are of interest. However, given the wide range of *β* values, each cow will require a different noise level to become sick, widening the distribution of *σ* values will produce a constant fraction of sick cows. In other words, if *β* is not limited to a single value, this will automatically enlarge the range of realistic *σ* values. The results of a model with such a uniform distribution are shown in Figure 5.

### Biological interpretation of the model

The model presented here contains four elements:

- linear bacterial growth (i.e. a constant term in the ODE).
- Destruction of the bacteria by the immune system.
- Supra-exponential bacterial growth (i.e. a supra linear term in the ODE).
- Random fluctuations.

The first term is expected in any model where bacteria grow with no saturation. Similarly the second term is expected in any model where bacteria are affected by the immune response of the host, including killing of bacteria by B or T cells. The two last terms are slightly more complex.

The non-linear bacterial growth can occur whenever existing bacteria facilitate the growth of more bacteria. In other words, there is a positive feedback of the current bacteria concentration on the future bacterial growth. The opposite may also happen, where bacterial growth prevents or reduces killing of existing bacteria. Such mechanisms are actually observed in MAP where bacteria organize in large granuloma and within these granuloma, they are protected against killing [23]. Moreover, cytokines secreted by infected cells limit the growth of active macrophages and reduce transition of macrophages to an activated macrophage. Such feedback loops are all expected to produce a non-linear growth rate.

The random fluctuations used here were multiplicative. In other words, random elements increase or decrease the net growth rate of the bacteria, either through a weakening of the immune response following other diseases or stressful events such as giving birth or transport events [24,25]. A similar random event may take place within the intestinal tract when conditions are suddenly very favorable.

## Discussion

From an evolutionary point of view, latency seems to be the optimal solution for pathogens, since it ensures the long term survival of their growth environment. Virulence can thus be treated as an accident of the pathogen life cycle. Indeed, many models were developed, to explain the emergence of virulence from an evolutionary point of view (e.g. [26-30]). However, the focus of these models was mainly on the evolutionary fitness advantage induced by virulence, and not the specific mechanism driving virulence. In parallel the issue of transition to AIDS in HIV was studied and multiple complex models were proposed [31,32], all having a common theme of a slow time scale inducing the long sub-clinical period between HIV infection and AIDS. Similar models were developed for Tubercle Bacillus (TB) [33].

The role of the immune system is crucial, with a quite general agreement that sub-clinical stages are basically induced by immune control, and that the transition to clinical disease is often associated with an escape from immune control [34-36]. Similar arguments have been raised in very different domains, such as the role of the immune response in tumor immune-surveillance and immune-editing [37,38].

However, a mathematical model of the basic mechanisms driving the exit from latency in general and methods to validate these conclusions at the epidemiological level are missing. We present here a comparison of multiple mathematical models, where the objective was to model the MAP shedding patterns. Ultimately, the model is gauged against observed within host immune dynamics on the observed MAP shedding patterns in cows from real-life populations. All models that were used contained a single variable (the bacteria). All other elements, such as the immune response, were assumed to be constant. Within these models, we show a model with two attractors: one representing the low bacteria concentration state and one representing the high shedding state. Stochastic transitions between these two attractors provided the simplest of the observed features, mainly a relatively flat probability of high shedding and clinical signs after an initial low or intermittent shedding period and a rapid transition to high shedding.

Other models could also be adapted to give similar results, but this would require more complex models and assumptions on the distribution of parameters. The two-state model presented here is far from being the only possible model in this category. In recent years many mathematical models have been studied that describe a wide variety of different systems: biological systems [39-41], physical systems [42-44], economic systems [45-47], etc. many of these models include two stable states [48-50]. The precise model to be used is of limited importance as long as the general probabilities to move from one state to the other are maintained.

While all models presented here are obviously overly simplistic, the objective of these models was to describe the essence of MAP within-host infection dynamics. More complex models may better reproduce many details of the dynamics, but will require many more, often unsupported, assumptions.

An important conclusion from these models is that the best method to prevent the transition to high shedding, in infected cows is to limit external events or other diseases. This can be checked by comparing the fraction of infected cows that develop clinical signs in different conditions.

A caveat of the proposed models is that they do not explicitly integrate the dynamics of the immune response. Thus, they cannot be directly compared to experimental observation on the relationship between immune response properties and MAP shedding patterns. A second caveat is the absence of early shedding in all models studied here. Infected calves are known to have an early shedding phase shortly after initial infection. However in all models studied here the development is unidirectional from non-infected to infected to high shedding. These two caveats can be solved using a model that includes the acquirement of a specific adaptive immune response following infection. However, there does not seem to be enough immuno-epidemiological observations at this stage to justify a more complex model.

While we here focused on MAP, the conclusions from this analysis are relevant to a large group of diseases with a similar epidemiology. We used a few criteria, such as the fraction of cows becoming high shedders, the dose response and within cow infection dynamics. It would be of interest to check if diseases can be broadly divided into groups fitting each type of model presented here.

## Abbreviations

AIDS: Acquired immune deficiency syndrome
BLV: Bovine leukemia virus
CFU: Colony-forming unit
ELISA: Enzyme-linked immunoSorbent assay
HCV: Hepatitis C virus
HIV: Human immuno-deficiency virus
HSV: Herpes simplex virus
MAP: Mycobacterium Avium subspecies Paratuberculosis
ODE: Ordinary differential equation
SDE: Stochastic differential equation
TB: Tubercle vacillus
